# Impact of Pathologic Complete Response following Neoadjuvant Chemotherapy ± Trastuzumab in Locally Advanced Breast Cancer

**DOI:** 10.1155/2021/6639763

**Published:** 2021-02-12

**Authors:** Taher Al-Tweigeri, Mahmoud Elshenawy, Ahmed Badran, Ayman Omar, Kausar Suleman, Osama Al Malik, Ihab Anwar, Noha Jastaniya, Asma Tulbah, Mohammad Al Shabanah, Dahish Ajarim, Adher Al Sayed

**Affiliations:** ^1^Medical Oncology Section, Oncology Centre, King Faisal Specialist Hospital and Research Centre, P.O. Box 3354, Riyadh 11564, Saudi Arabia; ^2^Clinical Oncology Department, Faculty of Medicine, Menoufia University, Shebin El Kom 32511, Egypt; ^3^Clinical Oncology Department, Faculty of Medicine, Ain Shams University, Cairo 11591, Egypt; ^4^Clinical Oncology and Nuclear Medicine Department, Suez Canal University Hospitals, Ismailia 41522, Egypt; ^5^Surgical Oncology Department, King Faisal Specialist Hospital and Research Center, P.O. Box 3354, Riyadh 11564, Saudi Arabia; ^6^Radiation Oncology Section, King Faisal Specialist Hospital and Research Center, P.O. Box 3354, Riyadh 11564, Saudi Arabia; ^7^Anatomic Pathology Department, King Faisal Specialist Hospital and Research Center, P.O. Box 3354, Riyadh 11564, Saudi Arabia

## Abstract

**Purpose:**

This study was designed to examine the relationship between breast cancer molecular subtypes and pathological response to neoadjuvant chemotherapy (NAC) ± trastuzumab, in locally advanced breast cancer (LABC).

**Methods:**

Female patients with LABC (T2–T4, N0–N2, and M0) who received neoadjuvant chemotherapy + trastuzumab if HER2+ subtype, followed by surgery and radiotherapy ± hormonal therapy, were identified. The primary endpoint was pathologic complete response (pCR) in the breast and axilla (ypT0/ypN0), with final analysis on disease-free survival (DFS) and overall survival (OS).

**Results:**

Six hundred eighty-one patients with a median age of 44 years, premenopausal: 70%, median tumour size: 7.0 cm (range 4–11 cm), stage II B: 27% and III A/III B: 73%, ER+/HER2−: 40.8%, ER−/HER2−: 23%, ER+/HER2+: 17.7%, and ER−/HER2+: 18.5%. Overall pCR (ypT0/ypN0) was 23%. The pCR rates based on molecular subtypes were ER+/HER2−: 9%; ER+/HER2+: 29%; ER−/HER2−: 31%; and ER−/HER2+: 37%. At median follow-up of 61 months, ER+/HER2+ and ER+/HER2− subtypes had the best 5-year DFS and OS; meanwhile, ER−/HER2+ and ER−/HER2− subtypes had the worst.

**Conclusion:**

Women with ER+/HER2− disease are the least likely to achieve pCR, with the highest rates in HER2+ and triple-negative subgroups. Degree of response is associated with OS; despite the comparatively higher likelihood of achieving pCR in ER−/HER2+ and triple-negative, these subgroups experience a survival detriment. We are consistent with the published data that patients who attain the pathological complete response defined as ypT0/ypN0 have improved outcomes.

## 1. Introduction

It has been established that locally advanced breast cancer (LABC) is an extremely heterogeneous disease that involves an extensive variety of biological phenomena. It is mostly diagnosed at advanced stages and has poor prognosis [1, 2]. LABC has continued to serve as a serious problem with adverse outcomes in spite of all the revolutionary advancements made in context of cancer biology and introduction of targeted therapy for treatment of cancer [1]. In general, treatment of LABC is performed by considering it as a whole cohort. Clinical outcome is not predicted accurately with the TNM stage. However, the prognostic information can be refined through addition of biological characteristics which also prove to be very helpful in choosing suitable systemic treatments.

According to the findings of the CONCORD, a research conducted in Europe and the data published by the National Cancer Database, 4% of European and 8.5% of American patients suffering from breast cancer have LABC [3]. The situation is even worse in developing countries since the rate of incidence is between 33–77% [4–7]. In our healthcare setting, around 25% Saudi women are diagnosed with LABC. As per the Surveillance, Epidemiology and End Result (SEER) data, individuals suffering from stages IIIA and IIIB of breast cancer demonstrate five-year survival rates of 52% and 48%, respectively [1]. A promising approach for the treatment of LABC patients is the neoadjuvant chemotherapy (NAC). pCR has turned into a well-known surrogate marker indicating good long-term outcomes. Highly reduced risk of relapse and death has been reported for patients who have achieved a pCR regardless of their molecular subtype and initial stage [5, 6, 8].

Accurate pathological analysis following NAC is of great importance since it is helpful for the prediction of prognosis and for determining the effectiveness of the treatment. Classification of breast cancer using immunohistochemical marker expression or gene expression array data has recently been recognized. During the 13th St. Gallen International Breast Cancer Conference 2013, a novel surrogate intrinsic subtype of breast cancer was suggested to distinguish luminal A, luminal B (HER2−/HER2+), HER2 enriched, and triple-negative disease. With insufficient gene expression data for these subtypes, determination of subtype of cancer for an individual using immunohistochemical staining has proven to be a good approach in context of NAC [7, 9, 10]. Breast cancer subtypes have been shown to be associated with odds of achieving pCR in a meta-analysis on patient data [11]. Reduced rate of pCR together with favourable prognosis has been reported for luminal A subtype which is described as ER+, HER2−, and low KI 67 proliferative index (≤20%). Conversely, HER2+ and triple-negative patients had increased rate of pCR. An association has been found between luminal B subtype and intermediate rate of response [12]. HER2-positive/hormone receptor negative and triple negative subtype patients have demonstrated variable survival with or without a pCR [13]. Around 70% patients having triple negative (TN) disease and 40% HER2+ patients develop residual invasive carcinoma following surgery despite the progress made in context of NAC.

Anthracycline-based regimens represent an important treatment component in the management of patients with early-stage breast cancer, with reduced breast cancer mortality by 20–30% based on recent (EBCTCG) meta-analysis. It suggested that anthracycline should be used for patients with TNBC regardless of nodal status or HER2−/ER+ with significant nodal involvement [14]. On the other hand, taxanes were found to have a major clinical activity in breast cancer and proposed even in patients with low grade, ER+ tumor which are thought to be chromosomally stable [15].

This research is aimed at investigating the association between pathological complete response and outcomes in different molecular subtypes of breast cancer following neoadjuvant chemotherapy ± trastuzumab.

## 2. Patient and Methods

### 2.1. Procedure and Data Collection

This study was approved by the Institutional Review Board (IRB). The study involved patients who were histologically diagnosed with unilateral locally advanced breast cancer (T2 ≥ 4 cm, T3 or T4, N0–N2, and M0) of noninflammatory nature and of stages II B, III A, and III B and who were given treatment using NAC approach during 2005–2014 at this setting through an electronic database which was prospectively maintained. Subjects included in this review were females who were diagnosed with LABC and were treated with NAC plus trastuzumab when HER2 positive and definitive surgery and locoregional radiotherapy ± hormonal therapy as per indication. Clinical and pathological data were collected including age, tumour size, histopathological subtype, hormone receptors (HR) and HER2 status, type of chemotherapy, number of given chemotherapy cycles, kind of surgery executed, and condition on last visit. Diagnosis of invasive breast cancer was performed through true-cut needle biopsy. Immunohistochemistry of pretreatment biopsy was used to determine estrogen (ER), progesterone receptors (PR), and HER2. No subject having residual disease demonstrated repeated ER, PR, or HER2 postoperatively. When 10% or more, tumour cells were stained for ER and/or PR, and hormonal receptor (HR) was considered positive. IHC was conducted to determine HER2 status which was verified through fluorescent in situ hybridization (FISH). The status was taken to be positive when it was equivocal (2+) on IHC and when FISH ratio was more than 2. Clinical examination was conducted with caliper prior to every treatment cycle in order to evaluate the clinical response. As per the Response Evaluation Criteria in Solid Tumour (RECIST) version 1.1, the response was categorized as progressive disease (PD), partial response (PR), stable disease (SD), and complete response (CR). The opinion of the breast surgeon as well as the patient served to be the basis for the decision to execute breast conservative surgery (BCS) or MRM. Adjuvant endocrine therapy for at least five years is suggested for individuals having HR-positive tumours. In case of premenopausal females, tamoxifen or aromatase inhibitors + LHRH agonists were given, and in case of postmenopausal females, aromatase inhibitors are given. Adjuvant radiotherapy was administered to individuals treated with BCS. Patients demonstrating pathological positivity of four or more axillary lymph nodes or those demonstrating at least clinical stage III disease or tumour size ≥5 cm at the time of diagnosis were treated with postmastectomy radiotherapy. pCR was defined as the complete absence of viable invasive tumour cells on pathologic examination in the breast and axillary lymph nodes, including surgical margins (ypT0 and ypN0). Patients with residual carcinoma in situ only were considered to have no pCR. Individuals demonstrating residual carcinoma only in situ were said to exhibit no pCR. As per our institutional guidelines, a written informed consent was taken from all the patients prior to initiation of chemotherapy ± targeted therapy.

Four main chemotherapy approaches were used for patients who were grouped accordingly. First group was given with anthracycline alone (A no T) comprising of doxorubicin or epirubicin together with cyclophosphamide. Anthracycline and taxanes (T ± trastuzumab) were given to the 2^nd^ group, and this system comprised of epirubicin/cyclophosphamide after which docetaxel or weekly paclitaxel ± trastuzumab was given doxorubicin/cyclophosphamide after which docetaxel or weekly paclitaxel ± trastuzumab was given. Anthracycline followed by platinum-based chemotherapy (A-P) was given to the 3^rd^ group. This approach made use of epirubicin or doxorubicin after cisplatin/docetaxel ± trastuzumab. Taxanes alone (TC with no A) were given to the 4^th^ group, and this comprised of docetaxel/cyclophosphamide ± trastuzumab. Seventeen cycles of trastuzumab were administered to HER2-positive patients.

### 2.2. Study Endpoints and Statistical Analysis

The primary endpoint of this study was to determine the rate of pCR according to molecular subtypes.

The secondary endpoints were to determine DFS (defined as the interval between the date of surgery and the date of disease recurrence or death before recurrence). Disease-free patients were censored at the last follow-up date. OS is defined as the interval between the date of diagnosis and the date of death from any cause. Surviving patients were censored at the last follow-up date. The pCR rate was calculated for each molecular subtype, and the Fisher exact test was used to evaluate the relationship between the subtype and the pCR rate. Descriptive statistics were calculated using the median and the range for continuous variables and compared among different studying groups using the Wilcoxon test. Categorical variables, frequencies, and percentages were calculated and compared among different groups using the chi-squared test. The probabilities of OS and DFS were calculated using the Kaplan–Meier estimator. Survival curves were compared using the log-rank test. *p* value <0.05 was considered significant. Statistical analysis was applied using SPSS version 20.

Overall survival (OS) and disease-free survival (DFS) were correlated with the kind of NAC used and various molecular subtypes including ER+/HER2−, ER+/HER2+, ER−/HER2+, and ER−/HER2−.

## 3. Results

### 3.1. Enrollment and Demographics

**Figure 1:**
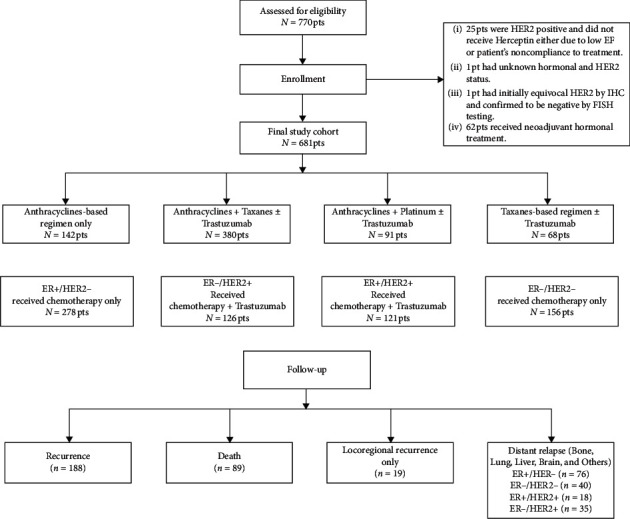
Consort diagram depicting the entire study cohort.

Three thousand and two hundred forty patients were seen at our oncology centre during the period between January 2005 and December 2014. 770 (25%) patients had LABC, and 625 (19%) had stage IV disease. 89 patients from the 770 LABC patients were excluded.  Figure 1 presents a flow diagram of the patient cohort. As per the criterion of this study, 681 cases were reviewed.  Table 1 shows the baseline clinical features of the population of this study. The median age of 44 year, and the majority of the patients were premenopausal (70%), stages III A and B (73%), ER+ HER2− (40.8%), ER− HER2− (23%), ER+ HER2+ (17.7%), and ER− HER2+ (18.5%). BCS and MRM were performed in 12% and 88%, respectively.

### 3.2. Efficacy

All patients demonstrated a clinical response during a follow-up of 38–92 months (median, 61 months). Out of 274 HER2+ individuals, pCR was achieved in 82 (33%). Conversely, 74 (17%) out of 434 HER2− patients achieved pCR (ypT0/ypN0). Overall pCR was found to be 23%. There was a significant difference in the achievement of pCR in different subtypes. 37% ER−/HER2+, 31% ER−/HER2−, 29% ER+/HER+, and 9% ER+/HER2− achieved pCR.  Table 2 summarizes pathologic responses of patients receiving NAC. On the basis of chemotherapy regimens ± trastuzumab, subjects were categorized into four groups. As shown in Figure 1, A + T, A no T, A + platinum, and T no A were given to 380 (56%), 142 (21%), 91 (13%), and 68 (10%) patients. Pertuzumab was not recommended for any patient. Likewise, genetic testing was not conducted for triple-negative subjects. Data relating to suitability or preference of the patients for breast-conserving surgery was not collected owing to small rate (12%) of this surgery in this population.

### 3.3. Survival Analysis

The overall recurrence rate (locoregional and systemic) was 28% (*n* = 188) (90% (*n* = 169) had distant recurrence and 10% (*n* = 19) relapsed locally); of those, 68% (*n* = 127) was HER2 negative and only 32% (*n* = 61) was HER2 positive. Rate of recurrence was the highest (45%, *n* = 76) in ER+/HER2−, and it was 23.6% (*n* = 40) and 20.7% (*n* = 35) in ER−/HER− and ER−/HER2+, respectively. The lowest recurrence rate (10.6%, *n* = 18) was noticed in ER+/HER2+.

Individuals having residual tumour postoperatively mostly demonstrated relapses. The brain, liver, lungs, and bones were the most common metastatic sites in HER2− patients. In HER2+ patients, the lungs, brain, bones, and liver were affected together with leptomeninges. 89 (13%) patients died because of progression of disease.

**Figure 2:**
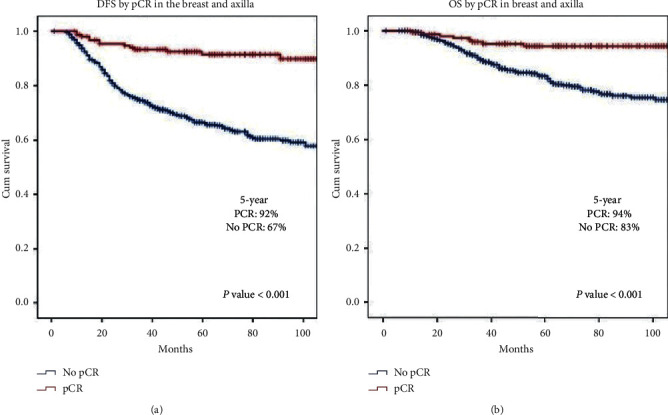
(a) Disease-free survival (DFS) and (b) overall survival segregated by pCR in the breast and axilla.

Figures 2(a) and 2(b) present the Kaplan–Meier survival determination as per pCR. In comparison to subjects with no pCR, subjects with pCR demonstrated a significant correlation with DFS and OS. Five-year DFS was better (92% vs. 67%) in case of patients with pCR in comparison to those with no pCR as shown in Figure 2(a) (*p* value <0.001). Patients with no pCR demonstrated 83% five-year OS and patients with pCR (ypT0/ypN0) demonstrated 94% five-year OS as shown in Figure 2(b) (*p* value <0.001). This study also involved a survival analysis conducted following exclusion of patients with pCR for drawing comparison of DFS demonstrated by different subtypes of patients having residual disease. Five-year DFS for ER−/HER2+, ER+/HER2+, ER−/HER2−, and ER+/HER2− patients were 52%, 77%, 61%, and 7%, respectively (Table 3). It shows poor DFS for patients with residual disease irrespective of the molecular subtype. Our data suggest that pCR (ypT0/ypN0) has decreased the risk of relapse and death differences with a statistical significance (*p* value = 0.001). Yet, differences in OS and DFS in treatment groups were statistically insignificant. Individuals received anthracycline-based regimen (*n* = 142) demonstrated 70% five-year DFS. Likewise, 5-year DFS was 71% (*n* = 380), 76% (*n* = 91), and 78% (*n* = 68) in case of anthracycline and taxanes, anthracycline and platinum, and taxane-based regimen, respectively.

**Figure 3:**
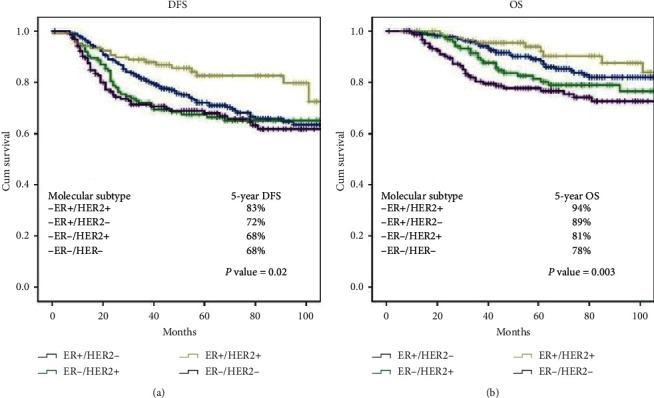
(a) Disease-free survival (DFS) and (b) overall survival segregated by breast cancer molecular subtypes.

Significantly different DFS was found in different subtypes. Highest five-year DFS (83%) was found in ER+/HER2+ in comparison to 72%, 68%, and 68% five-year DFS in ER+/HER2−, ER−/HER2+, and ER−/HER2− (Figure 3(a), *p* value, 0.02). Five-year OS was found to be 94%, 89%, 81%, and 78% in ER+/HER2+, ER+/HER2−, ER−/HER2+, and ER−/HER2− as shown in Figure 3(b).

## 4. Discussion

This study was aimed at analysis of the predictive and prognostic accuracy of molecular subtypes in females with LABC who were subjected to neoadjuvant chemotherapy ± trastuzumab. Molecular subtype was found to be a helpful tool in prediction of pCR. High pCR rate together with distinct survival outcome were demonstrated by triple-negative and HER2+ patients in comparison to luminal subtype. Response rates of 58% and 91% after neoadjuvant chemotherapy have been reported by earlier studies with 3–62% pCR [13–29]. However, subjects of these studies were not LABC patients only. Since this research involved 681 LABC patients, it is amongst largest studies investigating the association between molecular subtypes and responses to NAC. Variable NAC sensitivities were exhibited by different subtypes. Highest pCR rates were observed in ER−/HER2− and ER−/HER2+ and the lowest pCR rate by ER+/HER2−. Hence, the molecular subtype was proved as a strong independent predictor of OS and pCR. Achievement of pCR correlated with improved 5-year survival in all subtypes (Figures 2(a) and 2(b), Table 3)

In context of neoadjuvant therapy, achievement of pCR has been found to be strongly correlated with improved event-free and overall survival (EFS&OS) [13, 30]. Cortazar et al. conducted a pooled analysis involving 12 NAC trials (11955 patients) and reported that pCR in axilla as well as breast was more strongly linked with improved OS and EFS as compared to pCR in breast only. Moreover, patients with pCR exhibited 56% reduction in recurrence risk as compared to those with residual disease. pCR can therefore be regarded as a promising prognostic tool with 71%, 92%, and 84% reduction in death risk in ER+/HER−, ER−/HER2+ and ER−/HER2− subtypes. pCR rate of 7.5% has been reported by CTneoBC study involving analogous luminal population (ER+/HER−) which is comparable to population of this research. pCR rate of TN patients (33%) was quite comparable to our finding (31%). The pCR rate of HER2+ patients was higher for ER− instead of ER+ tumour (50% vs. 31%) in comparison with our data (37% vs. 29%), respectively. These pCR rates were comparable and, however, lesser than CTneoBC research [31].

During phase II trial of neoadjuvant (FEC100) after which cisplatin/docetaxel together with trastuzumab was given to HER2+ LABC patients, we found pCR rates in ER−/HER2+ and ER+/HER2+ to be 62% and 56%, respectively. Only 36% pCR rate was exhibited by TN patients. This treatment approach is included in the institution's guidelines with pertuzumab being added recently for treating HER2+ and TN subtypes [16].

Gentile et al. have also analysed responses of LABC patients towards NAC and found variable rates of pCR in different molecular subtypes. Overall pCR rate was found to be 25%. pCR rates for ER+/HER2−, HER2+, and TN patients were found to be 7%, 48%, and 23%, respectively. Increased pCR demonstrated by HER2+ patients could be because of utilization of pertuzumab. However, patients in this study had disease of more advance stages, and the median tumour size was 6 cm. Moreover, the median age was 52 years. These studies indicate that the response to chemotherapy ± targeted therapies is not dependent on the tumour size. In fact, tumour biology affects pCR [31]. This research has validated the finding that patients with pCR exhibit desirable outcome [32].

During this research, patients with pCR demonstrated better five-year DFS. In particular, five-year DFS in patients with pCR was found to be 92% in comparison with 67% in patients without pCR, thereby making the difference between the two as 25% (P < 0.001). Moreover, poor DFS was found in patients with residual disease irrespective of the molecular subtype. It implies that absence of response towards NAC indicates unfavourable outcomes in all subtypes. Reports indicate association between luminal A tumours and favourable survival outcomes in comparison with other subtypes. Conversely, TN and HER2+ patients exhibited most unfavourable outcomes [32, 33].

6094 females with invasive breast cancer were analysed through the Surveillance Epidemiology and End Result cancer registry (SEER) data, and it was found that molecular subtypes affected the four-year breast cancer specific survival. OS pattern was best in ER+/HER2+ patients, followed 20 in ER+/HER2− and ER−/HER2+ patients. Triple-negative patients exhibited worst OS [34]. Spring et al. recently conducted a meta-analysis involving 52 studies with 27,895 subjects for investigating the value of pCR after NAC. Overall pCR was found to be 21%. pCR was 36%, 33%, and 9% in HER2+, TN, and ER+/HER− patients, respectively. Examination of association between pCR and survival rate revealed that five-year EFS for patients with pCR was 88%, and it was 67% for patients without pCR. Moreover, five-year OS in patients with pCR was 94%, and in patients without pCR, it was 75%. Five-year EFS and OS varied in different subtypes. Five-year OS for ER+ patients with pCR was 98%, and for ER+ patients without pCR, it was 82%. Likewise, in case of HER2+ patients, five-year OS was 86% and 63% in patients with pCR and without pCR, respectively. In case of TN patients, five-year OS was 84% in patients with pCR and 47% in patients without pCR. Relationship between pCR and improved EFS was comparable in patients treated with adjuvant chemotherapy and those who were not treated with it. Findings of this meta-analysis point towards the fact that pCR can serve to be a strong surrogate endpoint for HER2+ and TN patients [35]. Intriguingly, our results were in agreement with these data as shown in Figure 3(a) and (b) and Table 3. During this research, the relapse rate was highest (45%) in ER+/HER2− patients followed by ER−/HER− (23.6%) and ER−/HER2+ (20.7%). ER+/HER2+ demonstrated lowest relapse rate (10.6%). A majority of relapses were seen in individuals who exhibited residual disease postoperatively. Similarly, earlier studies have reported variable rates of relapses in different individuals having different molecular subtypes. Differences in timing and pattern of relapses have also been reported in different molecular subtypes. TN patients relapsed within initial 3–5 years of the disease and the risk reduced to marginal following this period. Relapse risk was persistent and was five years in ER+ patients [32, 33, 36]. During this research, 30% ER+/HER2− patients relapsed persistently following 5 years. However, others relapsed during initial 2–5 years. Recurrence risk in HER2+ patients was considerably lesser than other subtypes possibly because of the fact that trastuzumab was given to all subjects included in this study. The pCR rates determined in this research are comparable with previously reported rates and greater for TN and ER−/HER2+ in contrast to ER+/HER2− patients. However, over 65% of HER2+ and 70% of TN patients had residual disease when their surgery was performed. These patients could be treated with adjuvant capecitabine (CTRATE-X trial) or trastuzumab emtansine (KATHERINE trail) [37, 38].

Correlation between pCR and improved OS and DFS (Table 3) is in agreement with earlier reports. Researchers are examining numerous approaches in postneoadjuvant setting like targeted therapy and immunotherapy. Clinical trials investigating treatment of residual disease can benefit from detailed information relating to genomic and molecular mechanisms. Technological advancements made in the field of biology during past years such as DNA sequencing, field-resolved infrared spectroscopy, and acquisition of gene expression profiles have improved our knowledge relating to tumour biology, development, and progression of tumour and drug resistance [39, 40].

Database of our institution proved to be very helpful in investigating this crucial aspect of LABC. Besides the retrospective design, this research has other limitations as well. Results could have been affected by unknown biases. KI-67 was not available for more grouping. The researchers did not consider the nature of adjuvant hormonal therapy, duration, and agents which could have influenced the OS. Though molecular subtypes indicated OS independently, numerous prospective clinical trials have not seen such trend.

## 5. Conclusions

Responses to NAC and outcomes of patients with different subtypes of LABC differed substantially. Therefore, molecular subtype can be regarded as strong independent predictor of pCR and OS. Improved five-year survival was seen in patients with pCR irrespective of the subtype. In comparison to ER−/HER+ patients, ER+/HER2+ patients exhibited improved five-year OS and DFS and lesser rate of relapse. ER+/HER+ patients with pCR did not show relapse at all. pCR (ypT0/ypN0) can serve as promising surrogate endpoint for TN and ER−/HER2+ patients but not for ER+/HER2− patients. Residual cancer burden (RCB) index may serve as a substitute surrogate endpoint for these patients after neoadjuvant setting serves as an opportunity for adjuvant clinical trials for testing novel drugs in populations with high proportions of high-risk individuals with residual tumour following neoadjuvant therapies.

## Figures and Tables

**Table 1:** Baseline demographic and clinical characteristics of the study population (*N* = 681).

Characteristics	No.	%
Age (years)		
Median	44	
Range	(38–60)	

Menopausal status		
Premenopausal	477	70
Postmenopausal	194	27

Clinical tumour stage		
II B	185	27
III A	215	32
III B	281	41

Clinical (T) stage		
T2	178	26
T3	246	36
T4	257	38

Breast primary tumour size (cm)		
Median	7 cm	
Range	4–16 cm	

Histology		
IDC	681	100

Histological grade		
Grade II	318	47
Grade III	325	48

Type of breast surgery		
Mastectomy	599	88
BCS	82	12

Category of NAC		
Anthracycline-based alone	142	21
Anthracycline and taxanes based	380	56
Platinum based	91	13
Taxanes based only	68	10

Hormonal receptor status		
ER+/PR+	315	47
ER+/PR−	84	12
ER−/PR−	275	41

Molecular subtypes		
ER+/HER2−	278	40.8
ER−/HER2−	156	23
ER+/HER2+	121	17.7
ER−/HER2+	126	18.5

HER2 status by IHC		
Negative	434	64
Positive	247	36

No., number; IDC, invasive ductal carcinoma; BCS, breast-conserving surgery; NAC, neoadjuvant chemotherapy; ER, estrogen receptors; PR, progesterone receptors; HER, human epidermal growth factor receptor 2; IHC, immunohistochemistry.

**Table 2:** Pathologic complete response rates in different breast cancer molecular subtypes.

Response	pCR breast (%)	pCR axilla (%)	pCR breast and axilla (%)
Total HER2 negative (*n* = 434)			
ER+/HER2− (278)	17	31	9
ER−/HER2− (156)	37	60	31

Total HER2 positive (*n* = 247)			
ER+/HER2+ (121)	38	50	29
ER−/HER2+ (126)	52	48	37

Total HER2 negative and positive (*n* = 681)	32	44	23

pCR, pathological complete response; ER, estrogen receptors; PR, progesterone receptors; HER, human epidermal growth factor receptor 2; IHC, immunohistochemistry.

**Table 3:** Summary of 5 years survival segregated by intrinsic subtypes.

	ER+, HER2− (%)	ER−, HER2− (%)	ER+, HER2+ (%)	ER−, HER2+ (%)
DFS				
psCR	92	83	100	93
No pCR	70	61	77	52

OS				
pCR	96	91	100	93
No pCR	88	72	91	74

ER, estrogen receptors; PR, progesterone receptors; HER, human epidermal growth factor receptor 2; pCR, pathological complete response; DFS, disease-free survival; OS, overall survival.

## Data Availability

The data used to support the findings of this study are available from the corresponding author upon request.

## Abbreviations

NAC: Neoadjuvant chemotherapy
LABC: Locally advanced breast cancer
pCR: Pathologic complete response
BCS: Breast-conserving surgery
MRM: Modified radical mastectomy
SEER: Surveillance, epidemiology, and end result
TN: Triple-negative
IRB: Institutional Review Board
HR: Hormone receptors
ER: Estrogen receptors
PR: Progesterone receptors
IHC: Immunohistochemistry
FISH: Fluorescent in situ hybridization
CR: Complete response
SD: Stable disease
PR: Partial response
PD: Progressive disease
RECIST: Response evaluation criteria in solid tumours
DFS: Disease-free survival
OS: Overall survival
REC: Research Ethics Committee.
